# Understanding drug exceptional access programs (DEAPs) in Canada, and their associated social and political issues

**DOI:** 10.1186/s12910-024-01038-8

**Published:** 2024-03-26

**Authors:** Pierre-Marie David, Kayley Laura Lata, Marie-Eve Bouthillier, Jean-Christophe Bélisle-Pipon

**Affiliations:** 1 Université de Montréal, Faculty of Pharmacy, 2940 Chem. de Polytechnique, Montréal, QC H3T 1J4 Canada; 2 Faculty of Law (LL.M. Candidate), McGill University, 3644 Peel St, Montreal, QC H3A 1W9 Canada; 3 McGill Department of Equity, Ethics and Policy, 2001 McGill College Avenue, Montréal, Québec H3A 1G1 Canada; 4 CISSS Laval Ethics Center, 1755 boulevard René-Laennec, Laval, QC H7M 3L9 Canada; 5 Université de Montréal, Faculty of Medecine, 2900 Edouard Montpetit Blvd, Montreal, QC H3T 1J4 Canada; 6 Simon Fraser University, Faculty of Health Sciences, Blusson Hall, Room 11300, 8888 University Drive, Burnaby, BC V5A 1S6 Canada

**Keywords:** Exception, Gene therapies, Canada, Access, Pharmaceuticals, Equity

## Abstract

Drug exceptional access programs (DEAPs) exist across Canada to address gaps in access to pharmaceuticals. These programs circumvent standard procedures, raising epistemic, economic, social and political issues. This commentary provides insights into these issues by revealing the context and procedures on which these programs depend.

## Introduction

New cancer drugs pose numerous problems of access and health equity in many countries and across Canadian provinces. The case of pertuzumab as a neoadjuvant in breast cancer treatment has been highlighted as revealing such inequities between Canada and other countries, as well as within Canada itself, where Québec’s population would have access to this antibody, while a negative evaluation from the Canadian Agency for Drugs and Technologies in Health (CADTH) would make it inaccessible in the rest of the country [1]. This case provides an opportunity to clarify the health and equity issues associated with exceptional access programs, as well as pharmaceutical policies on this matter. After describing the specific case of pertuzumab, we will see how it reveals a little-questioned object of research: drug exceptional drug access programs (DEAPs).

Recent pharmaceutical industry development towards immunotherapies, gene therapies and more broadly personalized medicine have led these DEAPs to play a key role, allowing access to personalized treatments under conditions not yet approved, thus creating a form of parallel market. The pharmaceutical industry’s access programs, in the form of patient support programs or compassionate programs, which can be understood as DEAPs, are beginning to be studied and understood, but they remain very flexible and difficult to grasp [2]. Our interest here in this commentary is to refer more specifically to public DEAPs, without directly considering manufacturers’ support programs, which pose other specific problems. The programs and dynamics surrounding exceptional access to pharmaceuticals are underdocumented. How do these programs work in practice? What are the health and political issues generated by DEAPs in the era of gene therapies, precision medicine, and ultra-expensive therapies? Addressing these issues requires delving into the links between pharmaceutical exception programs and the new economic and scientific dynamics of pharmaceutical development.

## Controversy over pertuzumamb as neoadjuvant

In December 2022, a commentary in *Current Oncology* identified inequities in access to neoadjuvant partuzumab in Canada [1]. Rayson and colleagues first identified inequities in access resulting from varying assessments between states. More surprisingly, the commentary also highlighted inequities within Canada, and the fact that Québec has access to the drug unlike other Canadian provinces. Which is not quite the case. The case of pertuzumab as a neoadjuvant provides an opportunity to clarify the issues involved in current programs for exceptional access to drugs, as well as pharmaceutical policies on this matter in Québec.

Pertuzumab, marketed as Perjeta by Hoffman-La Roche, has been positively evaluated by several regulatory authorities outside Canada as a neoadjuvant cancer therapy. CADTH, however, has given the drug a negative review in 2022 for these same indications. This poses several access problems for Canadian patients, who find themselves short-changed, or have to pay out-of-pocket or through their private insurance for this drug. In February 2022, the Québec health evaluation institute INESSS (*Institut national d’excellence en santé et services sociaux*) positively recommended this drug for this very indication, but with economic conditions. This means that the Minister of Health must negotiate the price to allow definitive access. Since February 2022, the Minister has been negotiating with Hoffman-La Roche, the company that markets this molecule. The drug is therefore not officially available to Québec women, as suggested by Rayson et al. 2022. Nevertheless, Québec does have an exceptional program for access to drugs for “Particular Medical Necessity”, a category through which pertuzumamb as a neoadjuvant has been accessed by patients in Québec.

In fact, pertuzumab is only accessible in Québec within healthcare facilities (e.g., hospitals), via a doctor’s request for “Particular Medical Necessity” (NMP, nécessité médicale particulière, in French). This mechanism for exceptional access to the drug dates to a 2015 law reforming the organization of the healthcare system in Québec, which was clarified in 2016, in a circular specifying the guidelines for processing these NMP requests. Generally, hospitals set up review committees for these requests. These committees are made up of various experts whose role is to give an opinion to the Hospital board of professionnals (CMDP, Comité des Médecins, Dentistes et Pharmaciens, in French), which will ultimately decides whether to grant access to these drugs that are not on the approved drug lists. In Québec, this is a decentralized process: each establishment organizes itself and makes these decisions on its own. The question of access to pertuzumab reveals the health policy issues raised by exceptional drug access programs, and the potential inequities they may raise.

## Difficulties in defining drug exceptional access programs across Canada

### In Québec

For many years, Québec has been a privileged territory for the pharmaceutical industry. With notable innovations, these privileges were reinforced in the 2000s as pharmaceutical policies were very advantageous for the industry [3]. In 2007, for example, the Québec Ministry of Health adopted a Drug Policy [4] with four main objectives: accessibility to drugs, establishment of a fair and reasonable price for drugs, optimal use of drugs and maintenance of a dynamic biopharmaceutical industry in Québec. This last objective is indicative of a specific conception of health as an economic driver through pharmaceutical industry sector by the government. This policy also allowed for an open door from the industry to the Ministry of Health. One of the concrete effects of this policy for pharmaceutical regulation is the acceleration of drug evaluation procedures. While regulation is still perceived as a limiting step in reaching the market, the evaluation of a drug can now be initiated by the INESSS before even obtaining the approval of Health Canada. In this context, exception programs become outlets for these innovations. Four DEAPs have been implemented in Québec (see Table 1): (i) the “Exceptional Drug Program,” (ii) the “Exceptional Patient Program,” (iii) the “Particular Medical Necessity” program which gives access to non-listed treatments in health care institutions but with federal compliance and (iv) the “Exceptional Treatments Program” for non-listed drugs without federal compliance [5, 6]. Some aim at controlling price and access, while others are used in hospital settings in a manner that bypasses the standard procedure set by provincial and hospitals’ lists. In addition, some compassionate programs and patient support programs [7] can also be considered DEAPs, as is the case in Québec.

**Table 1:** Drug exceptional access programs in Québec

	Exceptional drug program (“Médicament d’exception”)	Exceptional patient program (“Patient d’exception”)	Particular medical necessity (“Nécessité médicale particulière”)	Exceptional treatment (“Traitement d’exception”)
Health Canada notice of compliance	Yes	Yes	Yes	No
Listed on the provincial public insurance list	Yes	Yes	No	No
INESSS conditions	Only if therapeutic indication recognized by INESSS and with exceptional reimbursement	Use associated with patient specific conditions	Unevaluated or evaluated negatively	Unevaluated
Decision	Régie Assurance Maladie du Québec (RAMQ)	RAMQ	Hospital board of professionals (CMDP)	Hospital board of professionals (CMDP)
Access	In pharmacies and hospitals	In community and hospitals	In hospitals	In hospitals

### In Canada

Because of the distribution of legislative powers, the federal government funds part of the provinces’ health expenditures and authorizes drug marketing (through Health Canada). Provincial governments are responsible for ensuring the appropriate use of and access to drugs, which are generally the ones listed for reimbursement by provincial public insurances. This situation results in variability in the access to drugs [8] as well as equity issues [9, 10].

First, the federal “Special Access Program” provides exceptional access to non-approved drugs. This could be named a/the federal DEAP. We could also acknowledge that there are various barriers in providing pharmaceuticals through federal DEAP as demonstrated by the continuing lack of access to pharmaceuticals for certain populations such as indigenous people requiring tuberculosis drugs [11]. In the provinces, if the drugs are approved by Health Canada, but not listed by the ministries of health or used outside of their recommendations, their access is managed through provincial DEAPs. The actual names of these programs vary greatly within provinces and between them: “exceptional” (Ontario’s *Exceptional Access Program*), “compassionate” (*BC Cancer Compassionate Access Program*), “special” or “necessary” (Québec’s *Nécessité médicale particulière, BC Special Authority program*). Some provinces even use economic and moral language, labelling their program as “catastrophic” (Catastrophic Drug Program) [12]. Within the same province, different DEAPs co-exist, as presented in Québec, as well as in Canada. See Table 2.

**Table 2:** Other examples of drug exceptional access programs in Canada

	Federal: Special access program (SAP)	Pharmaceutical industry: Patient support programs (PSP) from pharmaceutical industry	Quebec : Particular medical necessity (“Nécessité médicale particulière »)	Ontario : Exceptional access program
Health Canada notice of compliance	No	Yes	Yes	Yes
Listed on the provincial public insurance list	No	Yes or No	No	No
use conditions	Not to encourage the early use of drugs	Determined by the PSP	Unevaluated or evaluated negatively	Unevaluated
Decision	Health Canada	Pharmaceutical industry	Hospital board of professionals (CMDP)	Executive officer ministry of health under advise of Committee to Evaluate Drugs
Access	In community and hospitals	In community and hospitals through PSP	In hospitals	In community and hospitals

There is great diversity and variability in the way these programs are organized, and access to the same drug can differ greatly from an inter-provincial/territorial basis (one province or territory to another) and an intra-provincial basis (e.g., between rural and urban settings, among different hospitals). Thus, within the same country, patients with the same needs are dependent on the DEAPs of their local settings for accessing the same drug. This raises major issues of equity in a context where the Canadian health care system is generally understood and praised for being equitable in terms of access, treatment, and structure. As a result, there is critical need for further examination, understanding and analysis/evaluation regarding equity issues and DEAPs. Furthermore, there is a need to consider the broader context of pharmaceutical development, as those programs are increasingly used to channel access to pharmaceuticals precisely designed in this specific context.

## Drivers of drug exceptional access programs

### The impulses of cancer treatments and their pharmacoeconomics

Successive generations of anti-cancer drugs have renewed hopes for clinical outcomes for both health professionals and patients. They act in an increasingly specific way on the specifics of cancers by offering a statistical prolongation of survival and sometimes a cure. In addition, they prohibit tumor growth for a period of time, and they can even reduce tumor growth. This ensures obvious clinical benefits: spending more time with loved ones, being able to adequately say goodbye to people around them or being present for significant moments such as a child’s wedding. In addition to the hope of a cure, health professionals and patients often perceive these considerations as compelling. Accordingly, their access to such programs is justified despite an unfavourable assessment of the treatment from a cost-effectiveness perspective.

In this context, the recommendations issued by agencies restricting access to anti-cancer drugs are largely contested by patients, patient associations and the pharmaceutical industry. They might lobby for off-label use, despite an unfavourable cost-benefit ratio. For the industry, anti-cancer drugs represent a very lucrative market and recent developments are pushing towards increasingly targeted (and therefore expensive) molecules. For patients and patient associations, the new anti-cancer drugs represent a hope of treatment often when other treatments have failed. Thus, they perceive this as a failure of the system not to try everything for a therapeutic hope where, in their view, the economic calculation no longer holds.

Following anti-cancer drugs, gene therapies have more recently represented the upward trend. The cost of these therapies is well beyond the ability of most third-party payers to pay. Gene therapies offer a cure for a health problem to a greater extent than most other drugs. This adds to their potency and attractiveness, which is often monetized at a selling price that goes far beyond the costs of research and development. These two classes of drugs often offer hope to patients, families, and clinicians. New waves of anti-cancer drugs and gene therapies provide renewed hope for patients who are at the end of their rope or facing a therapeutic dead end.

The impact of anti-cancer drugs on the health care system, especially in financial terms, has led to major institutional arrangements and a restructuring of the activities of agencies and third-party payers. Specific budgets have been set aside for access to cancer drugs, and agencies and third-party payers have set up dedicated review committees and separate processes for evaluating and managing access to cancer drugs. While these treatments hold great promise, they are not necessarily effective, let alone cost-effective (sometimes with modest impacts on DALYs and QALYs). Moreover, a specific range of cost by QALY has emerged to inform decision-making. Whereas a tacit threshold of 50 000$ per QALY has traditionally informed pharmaco-economic decisions, in the field of oncology this threshold might be doubled around 100 000$/QALY. Oncology seems to be connected to a specific form of valuation that has been coined as “malignant pharmacoeconomics” [13]. As a matter of fact, oncology drugs embody this exception with respect to both cost and regulation in many provinces in Canada.

### “Orphanization” and the quest for “nichebusters”

The pharmaceutical industrial model has long been based on a relatively small number of highly profitable products provided to vast populations, generating a significant share of pharmaceutical industry sales. These “blockbuster” drugs were expensive but not prohibitively so (for most insured people) and were largely reimbursed by public and private third-party payers. Most of these drugs were hugely profitable until their patent expiry. In the last decade, these traditional blockbusters have lost momentum, thus affecting sales and profitability of companies. Precision medicine (which can be defined as medicine seeking to improve patient stratification and management using biological information and biomarkers grounded on individualized therapeutic approaches to health [14], pharmaceutical developments and increasingly profitable drugs have transformed the marketing structure of the pharmaceutical industry. Pharmaceutical developments targeting more determinedly niche patients with products referred to by some as “nichebusters” have superseded mass production [15, 16]. As opposed to “blockbusters,” “nichebusters” have generated a business model characterized by highly expensive drug prices and a very small number of patients. The development of these “nichebusters” has fostered access and sustainability problems for public insurance systems. DEAPs seem an opportunistic solution for those problems.

This shift is driven by a multitude of factors, the most important of which is pharmaceutical development and innovation. This can be explained by the fact that the number of highly profitable products that can be sold on a large scale is decreasing. However, this is counterbalanced by an increased capacity for patient stratification, allowing better identification and grouping of patient needs and more precise mechanisms for meeting them. This is often shown to be beneficial, as it allows for increasingly personalized health care to be received. In the case of the drug nusinersen (brand name: spinraza), Wadmann and Hauge show precisely how Danish institutions have stratified patients and their disease to control costs while keeping access possible to certain eligible individuals. This strategy of “stratification” [17] leads to the fragmentation of patient populations, making them almost orphaned in their needs and from a therapeutic point of view.

These trends are driving changes in treatment categories. In recent years, orphan drugs, as they were categorized, have slipped from their initial definition. Until recently, orphan drugs were considered medicines that had limited market potential. To ensure safety, security (especially in terms of developing solid evidence despite far smaller populations), but also cost-effectiveness of this type of product, several jurisdictions such as the USA [18, 19] and the EU [20] have adopted a specific legislative framework for orphan drugs or rare disease treatments. The legislative frameworks also aim to stimulate the pharmaceutical industry, which had long argued that specific requirements represented unfair obstacles to the development of such drugs.

This definition was somewhat reversed when it became possible to target small groups of patients, not only for rare diseases but for the individualization (or clustering) of patients with specific health needs and calling for more expensive (and more profitable) products [21]. Since then, the pharmaceutical development model of orphan drugs has been replaced (at least in part) by new models emphasizing on stratification and individualization of the response to patients’ needs. Such a process can be labeled as “orphanization” since it entails that a drug, in its biotechnological development and marketing, seeks to target a very limited number of patients, making it an orphan drug. A major reversal, which can be attributed to the development of precision medicine, has thus taken place: an orphan drug is no longer a drug with limited marketing potential, but a drug with major marketing potential that is aimed at a limited number of patients [22].

This context invites us to rethink the exceptionality at the heart of DEAPs. Whereas they were once conceived as a means of ensuring access to medicines to meet the unmet demand of certain patients, DEAPs are now emerging in a more global economic and political context as ways of facilitating the supply of particular treatments. From this point of view, DEAPs are particularly well-suited to the contemporary context marked by the knowledge and economic developments of personalized medicine.

## Issues associated with DEAPs

This evolution of the pharmaceutical development and pharmacoeconomics sheds a new light on exceptional access-to-medicines programs and helps to formulate some of the epistemic, economic, social and political issues associated with them.

*Epistemologically*, DEAPs are not neutral regarding the knowledge considered and valued. As previously discussed, in the current pharmaceutical regime, the status of evidence is changing radically, considering smaller numbers of patients and decisions made sometimes more on phase II trials rather than on phase III research, requiring less patients but raising uncertainty. Secondly, the management enabled by DEAPs implies post-access monitoring in the form of Real-World Evidence, which is becoming a central tool in the governance of access. The risk of conducting fewer clinical trials is being overtly made by pharmaceutical companies, and in turn raised by patient groups. The relationship between access programs and the data and knowledge they promote must therefore be considered to fully understand the issues at stake.

*Economically*, the market for expensive orphan drugs is growing at an annualized rate of 32% and represents nearly 10% of Canadian pharmaceutical sales. Provincial regulatory agencies, such as Québec’s INESSS, acknowledge this expansion of the term “orphan”: “Products with orphan designations target rare diseases or small subpopulations of common “precision medicine” diseases and are likely to be highly expensive” [23]. Restructuring the locus of profitability from *block*busters to *niche*busters by stratifying patient populations (also called salami slicing strategy) and orphaning responses to health needs has laid the foundations for new pharmaceutical and therapeutic regimes, rationalising the high cost of otherwise low-cost or unapproved drugs. Thus, exceptional policies and practices might operationalize a tour de force by both facilitating access to drugs while rationing them at the same time. This operation is fruitful both for pharmaceutical manufacturers who are provided with a justification for their high prices, as well as policymakers who want to occupy both sides of the fence. A plausible explanation for the increased use of DEAPs is the new pharmaceutical regime and the growing pressure to bring promising drugs to the market quickly. Considering the ongoing difficulty in accessing expensive drugs and the trend towards a greater proportion of new drugs that have significant impacts on health insurers’ budgets, regulatory authorities may be led to evaluate such molecules negatively by specifying strict conditions. Thus, DEAPs are an important alternative means for the industry to reach the market, and for the government to provide access to expensive and uncertain drugs.

*Socially*, these programs generate relatively polarized reactions. Their proponents are often practitioners asking for the latest scientific developments to treat their patients (even if they have yet to be validated); or practitioners who want to use certain molecules for indications that have not been approved. Numerous patient associations, often supported by the pharmaceutical industry [24], are also lobbying for maintaining the concrete hope associated with access to certain drugs, even when efficiency and cost-effectiveness data are uncertain (or even unfavorable). It is the same logic that underlies industry sponsored compassionate programs deployed in hospitals. According to DEAP opponents, however, such programs (especially those sponsored by the industry) represent a form of strategic loyalty through compassion [25]. Indeed, programs sponsored by industry can provide drugs for other more regular DEAPs and make them more acceptable for health professionals as they do not draw from the common fund. Some practitioners and pharmacists are opposed to these programs and consider them unfair, as shown by our ongoing research in Québec. Offering drug access through exceptional programs entails circumventing the standard procedures for evaluating safety and effectiveness and jeopardizing public health insurance systems. Between these positions, policy-makers seem to remain hesitant. That said, underneath those perceptions of DEAPs lies important legal and political issues.

*Politically*, whereas the absence of a pan-Canadian policy is a major obstacle to equity in access to drugs [26–28], DEAPs contribute to address certain gaps in access to pharmaceuticals but they also create some of the access equity issues as they also contribute indirectly to the absence of a pan-Canadian policy. Indeed, Canada’s current patchwork of DEAPs adds to the patchwork of public or private drug plans. This has been shown to also expose households to considerable and inequitable financial risks, adding to the administrative costs of prescriptions. This situation also isolates the management of prescription drugs access from other key components of Canadian Medicare. The current debate on the reform of the Patented Medicine Prices Review Board (PMPRB) is also linked to issues associated with DEAPs. In particular, pharmaceutical companies have used Canadian access to medicines for rare diseases as leverage to oppose the changes [29]. Once we have better investigated and understood the political dynamics surrounding DEAPs, it will be important (even before having asked the question of their internal fairness) to know whether they do not contribute to preventing the development of a coherent Canadian pharmaceutical policy. Rather than denying access to important drugs, one alternative would be to think of DEAPs as part of a pan-Canadian pharmaceutical policy.

## Conclusions: understand DEAPs through the lens of equity

The literature on DEAPs lacks substantial analysis and significant data, as well as comparisons between the provinces and territories [30]. There is a need to document DEAPs and investigate how they work in theory, in addition to how they are used in practice and which actors and institutions are involved in the decision-making and intertwined through them. There is also a need to describe how patients have accessed the latest (often very costly) innovative therapies in different provinces and how the media has covered these issues. Whereas public decision-makers seem to remain hesitant to comment on DEAPs, mainstream media take stories about patients who are denied drug access very seriously, as well as the unaffordable prices set by pharmaceutical industries. In doing so, the media ends up framing public debate on very specific terms, at times invisibilizing more structural issues that go to the heart of public health insurance systems. In the absence of extensive and comparative knowledge of DEAPs, a pan-Canadian study is urgent and topical to generate data and to help develop a more critical discussion that is mindful of the legal and political issues associated with these programs.

## Data Availability

N/A.
